# Intestinal Infusoria in Man

**Published:** 1860-01

**Authors:** 

**Affiliations:** Stockholm


*Intestinal Infusoria in Man.* By Professor MALMSTEN, of Stockholm. Published originally in the *Archiv für Pathologische Anatomie*, and translated from the *Gazette Hebdomadaire*, by GEORGE S. BLACKIE, M. D.

CASE I.—A sailor, about thirty years old, entered the hospital of Seraphim on the 22d of March, 1856, suffering from a lienteric affection. He had had, two years before, cholera, which had been followed by various disorders of the digestive system, by constipation alternating with diarrhœa, etc. Frequently his meals were followed immediately by copious diarrhœa-like discharges, slightly colored, and containing a large amount of undigested food. A painful tenesmus, accompanied by an expulsion of small quantities of bloody fluid, supervened, when all the contents of the intestines had been passed by the stools. Then, to allay this, the patient was compelled to refill his stomach, which produced the same series of accidents. Enfeebled by the frequent exacerbations of the affection, he was finally obliged to confine himself to bed. He preserved always a remarkable appetite. The same symptoms continued to the 5th of April, in spite of the employment of numerous remedies. The skin was dry, but he had no fever, and the abdomen was not sensitive on being pressed. On examining the rectum, there was found near the anus an ulcer, with a hard base and swollen edges, covered with a purulent discharge. The microscopical examination of this liquid detected a large number of animalcules of the peculiar description given below. Cauterization with potassa fusa, and the injection of cod-liver oil, caused, at the end of a few weeks, the cicatrization of the ulcer.

The patient meanwhile took sulphate of quinine internally. He recruited his strengh, but the state of his digestive functions remained unchanged. At last, on examining the stools, immediately after their passage, with the microscope, the presence of an incredible number of infusoria was recognized,—infusoria similar to those which had been previously found in the pus which covered the ulceration of the rectum. These were found likewise in the mucus taken from the intestine by an instrument, and a great number of explorations and varied methods of examination produced but the same result.

M. Malmsten prescribed injections of chloro-hydric acid, which produced marked improvement. The number of stools was reduced to two a day, and when on the 28th of August the patient left the hospital, there was but a very small number of the animalcules in the evacuations. They were still more reduced in number when M. Malmsten saw his patient again in March, 1857.

The animalcules, very carefully examined by Professor Lowen, measured about a millemetre* in length, and had an oval form, which was considerably enlarged when they were full of food. They were well supplied wiht cilia around the buccal orifice, with which was continuous an œsophagus, prolonged in some instances by a dark line, indicating the presence of alimentary matters. The anus, situated at the posterior extremity of the body, was sometimes retracted, sometimes protruding. In the interior of the body one could see the contractile vesicles, an oval nucleus, and the food, such as grains of starch and particles of oil. These animalcules were very active in their movements; they died, moreover, some hours after quitting the intestine. On looking at these characters we find that they approach the paramœcia; hence M. Malmsten proposes for them the name, *Paramœcium coli.*

CASE II.—A woman, about thirty-five years old, presented herself, having the same symptoms as the subject of Case I.; the stools were generally darkened by the presence of blood, and the abdomen was very sensitive on pressure along the track of the colon. The infusoria were very abundant in the stools. The patient died of marasmus.

On a post-mortem examination, gangrenous ulcerations were found in the large intestines. The sanious pus which covered them contained the infusoria, but they were much more abundant on the mucus which covered the points not affected by disease, and especially the cæcum and vermiform process, which were healthy. None were found beyond the ileo-cæcal valve.

M. Malmsten supposes that the lienteric symptoms observed in these patients were due to an exaggeration of the peristaltic movements of the intestine and its secretions, a natural result of the irritation produced by the innumerable little parasites distributed over the mucous membrane of the colon. The two cases agree also in showing that the presence of these little beings is not necessarily owing to a diseased state of the intestine.

Is this a very rare disease, or entirely exceptional? M. Malmsten thinks it is neither. If it has not been seen up to this time by observers, it is undoubtedly because the infusoria die and disappear so very rapidly after their expulsion. It is necessary, then, in order to detect them, to examine the discharges as soon as possible.

M. Malmsten makes, further, the remark that these parasites, being exclusively found in the large intestines, permit of direct action on them by the use of injections; and he calls special attention to the advantageous effects obtained in the first case by the use of injections of chloro-hydric acid.—*Nashville Journal of Med. and Surgery.*

---

* Equal to 0.03937 of an English inch, or about two-fifths of a line.

